# Supplementary material for: Numerical simulation of the fine kinetics of dust reduction using high-speed aerosols
Source: PLoS One. 2023 Dec 27;18(12):e0286101. doi: 10.1371/journal.pone.0286101 (PMC10752516; doi:10.1371/journal.pone.0286101)
Supplement: Attachment — Submitted filename: Responses to Technical Check Results.docx [file pone.0286101.s001.docx]

Dear Editors and Reviewers:

Thank you for your letter and for the reviewers’ comments concerning our manuscript entitled “Numerical simulation of the fine kinetics of dust reduction using high-speed aerosols” (ID: PONE-D-22-33806). All comments are very important and they are great support for our scientific research work. We have studied comments carefully and have made correction **(Revised portion are marked in red in the text)**. The main corrections in the paper and the responds to the reviewer’s comments are as flowing:

**Reviewer 1**

The subject of the study is the single-particle collisional wetting process of micron-sized droplets on dust of different typical particle sizes in a high-speed airflow, which is investigated using a finite element-dynamic mesh modelling approach based on the three-phase flow theory. The reliability of the simulation is adequately given in terms of inter-particle forces, airflow streamlines and hydrophobic phenomena. A comparative study has been carried out to obtain the optimum wetting particle size ratio and collision angle for different typical particle sizes of dust. I think the results of the study can provide an important reference for the study of pneumatic spray capture of micron-sized particles. However, There are some minor problems with the article which should be slightly revised, I support the publication of this paper.

Answers: Thank you very much for the excellent and professional revisions of our manuscript. We have studied the reviewer's comments carefully and have made revisions that have been marked in red in the revised manuscript.

**Comment 1**)In this article, variables should be used in the correct format, page 10, paragraph 3 of the article.

Answers: Thank you for your comments.I have changed the variables to the correct format .( Page 10)

**Comment 2**)Three typical particle sizes of dust particles are selected for study in the article, and the reasons for selecting the particle sizes PM2.5, PM10 and PM20 for study.

Answers: Thank you for your comments.The necessary boundary conditions for numerical simulations are described in the text. Three types of dust, PM2.5, PM10 and PM20, were chosen because they are more hazardous to humans in the micron range and the differences between the three are more pronounced all being representative of the dust.

**Comment 3**)On page 19 of the article, heading 4.2, "At 1 µs, the relative motion velocity decreased to 1.5~2m/s." What does the relative motion velocity mean here.

Answers: Thank you for your comments. The relative velocity here refers to the relative velocity between the dust particles and the water mist particles after their collision, i.e. the difference in velocity between the two particles.( Fig.9 has been modified accordingly)

**Comment 4**)In heading 4.3 on page 20 of the article, select four specific c values Please explain the reasons for the selection

Answers: Thank you for your comments. Four specific c values are selected for four collision angles, corresponding to 0°, 15°, 30° and 45°, and the range of collision angles is divided into four equal parts, and then four representative values are selected to better analyse the influence of collision angle on wettability and to obtain conclusions

**Comment 5**)The relative velocity between gas and solid is 30m/s to 100m/s. What is the basis for choosing this velocity.

Answers: Thank you for your comments.The main simulation in this paper is the flow field of high speed air movement, and the velocity of water mist at the exit of the supersonic atomisation nozzle can reach about 50m/s, so the relative velocity between gas and solid is selected from 30m/s to 100m/s, mainly to study the dust trapping of water mist particles moving at high speed. The effect of relative velocity on the dust trapping mechanism is effectively studied through simulation

**Comment 6**)In this paper, the collision between a single droplet and a single dust is studied. it is suggested to change " droplets and dust particle" to " droplet and dust particle"

Answers: Thank you for your comments.I have changed the variables to the correct format .

**Comment 7**)The fonts in Fig. 13 and 14 should be adjusted to the same format.

Answers: Thank you for your comments.I have changed the variables to the correct format .( Fig. 13 and 14)

**Comment 8**)In heading 4.3 on page 20 of the article, select four specific c values Please explain the reasons for the selection

Answers: Thank you for your comments.The authors mainly study the collision of dust and water mist airflow in high speed movement. Although the collision of engineering dust and water mist airflow cannot reach a high relative velocity at present, the authors find the conclusion of dust reduction efficiency in the flow field of high speed airflow movement through simulation research, which provides the theoretical basis for future research and development of dust reduction equipment, the greater the relative velocity the better the wetting effect and increase the dust capture efficiency. It is believed that future on-site dust reduction equipment can reach the collision situation of the high-speed airflow field, and the authors will also conduct future research on dust reduction equipment based on theoretical studies. Special thanks to the reviewers for the questions raised, and the authors will conduct deeper research on the relevant contents in future studies.

Thank you very much for your suggestion!
